# Effects of a Forest Walk on Urinary Dityrosine and Hexanoyl-Lysine in Young People: A Pilot Study

**DOI:** 10.3390/ijerph17144990

**Published:** 2020-07-10

**Authors:** Ai Yamada, Yoshiko Sato, Tokushi Horike, Masamitsu Miyanaga, Da-Hong Wang

**Affiliations:** 1 Department of Biochemistry, Okayama University of Science, Okayama 700-0005, Japan; s18bm03ya@ous.jp (A.Y.); yo-sato@a.zaq.jp (Y.S.); miyanaga@dbc.ous.ac.jp (M.M.); 2 Wakayama Shin-ai Junior and Senior High School, Wakayama 640-8151, Japan; 3 Public Health Lab, Okayama 700-0973, Japan; horike@wing.ocn.ne.jp

**Keywords:** forest walk, dityrosine, hexanoyl-lysine, protein oxidation, lipid peroxidation, phytoncides

## Abstract

A few studies indicate exposure to forests may alleviate oxidative stress in the body. However, more evidence is needed to support this potentiality. The purpose of the current study aimed at examining whether there is any difference in urinary levels of oxidatively modified proteins or lipids—dityrosine (DT) and hexanoyl-lysine (HEL), respectively, after a forest or urban walk. The study was performed on 29 university students who took part in forest walks (Shinjo Village) in Okayama Prefecture of Japan and on 42 university students who took part in urban walks in the downtown area of Okayama City. Urine samples before and after the walks were analyzed for DT and HEL excretion. Air phytoncides during the walks were also measured. We found a decreased tendency in urinary DT and HEL (*p* < 0.05) in most participants after the forest walks, but not after the urban walks. We further found the total levels of air phytoncides in the forest field were 1.50 times higher compared with those in the urban field. This study suggests the possibility that regular immersion in a forest environment might contribute toward weakening of the oxidative modifications of proteins or lipids in the body.

## 1. Introduction

In the fields of preventive medicine and public health, much attention has been given in recent years to the effects of forest bathing (spending a few hours immersed in a forest environment. The forest environment here refers to a large area of land covered mainly by trees), such as increases in human natural killer (NK) activity and anti-cancer protein expression, decreases in blood pressure among hypertensive patients, and mental stress reduction [1,2,3,4,5]. However, the mechanisms underlying the health benefits of a forest environment are not well understood. It is known that oxidative stress is caused by a generation of excess reactive oxygen species (ROS) that exceed the capacity of antioxidant defense systems in the body to remove them. This imbalance may oxidatively damage DNA, proteins, and lipids [6,7]. Wang et al. recently reported that urinary hydrogen peroxide (H_2_O_2_) and 8-hydroxy-2′deoxyguanosine (8-OHdG) decreased after a forest walk but not after an urban walk [8]. Gibo et al. also found a decreased serum malondialdehyde-modified low-density lipoprotein level (MDA-LDL) after a forest walk [9]. On the other hand, Im et al. demonstrated that exposure to a forest environment for 2 h led to an elevation of serum antioxidant enzyme glutathione peroxidase [10]. This evidence probably suggests that exposure to a forest may play a role in alleviating oxidative stress and regulating the imbalance between the production and neutralization of ROS in the body [8]. However, more evidence is needed to support this potentiality.

Urinary dityrosine (DT) and hexanoyl-lysine (HEL) are known as deleterious products generated by ROS action in the body [11,12]. Many scientists have suggested they are sensitive biomarkers for detecting oxidation of proteins and lipids, respectively [13,14,15,16]. However, there was no report on how the levels of urinary DT or HEL change after a forest walk. The present study aimed at examining whether there is any difference in the levels of oxidatively modified proteins (DT) or lipids (HEL) in urine after a forest or urban walk. 

## 2. Subjects and Methods 

### 2.1. Study Design and Procedure

**Figure 1:**
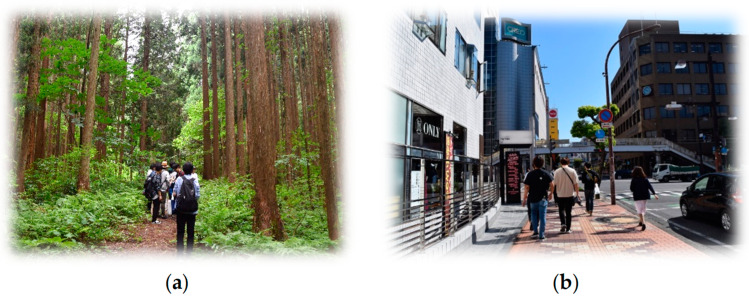
Pictures of the forest walk (**a**) and urban walk (**b**).

We carried out the forest walks in June, 2017–2019 in Shinjo Village (total area: 2.96 km^2^ located in northern Okayama Prefecture, Japan (Figure 1a), in which 45% of the trees are conifers, such as Japanese cypress (*Chamaecyparis obtuse* ((Siebold & Zucc.) Endl.) and Japanese cedar (*Cryptomeria japonica* (L.f.) D. Don), and 55% are broad-leaved trees, such as Japanese beech (*Fagus crenata* Blume). The Shinjo Village is one of the certified Forest Therapy Bases in Japan, where there is a 2 km long walking path used as a therapy course where you can take a forest bath. All participants accompanied a lab staff member and went there by chartered bus (about 2 h from Okayama City to Shinjo Village). The urban walks took place in July–October, 2016–2019 in the downtown area of Okayama City (Figure 1b), which has a population of 720,066 and a total area of 789.95 km^2^; the general traffic amount is less than 30,000 cars and trucks/24 h). Each walk comprised 2 h of slow walking with a gait velocity of less than 60 m/min [17], during which there were two to three short breaks as described by Wang et al. [8]. The urban walks were carried out in 2016–2019, and the forest walks in Shinjo Village were added to the study in 2017–2019 (Table 1). 

The studies were performed on 29 university students (male: 22, female: 7) who took part in the forest walks voluntarily, including 11 students in 2017, 11 students in 2018, and 7 students in 2019; and on 42 students (male: 31, female: 11) who took part in the urban walks, including 10 students in 2016, 12 students in 2017, 11 students in 2018, and 9 students in 2019 (Table 1). The studies carried out in 2017–2019 had a cross-over design and all participants (ages 21–23) in each year attended both the forest and the urban walks in one group, except 3 absentees from the forest walks. A self-reported questionnaire collected participants’ data on cigarette smoking, alcohol consumption, exercise/physical activity, and vegetable/fruit intake. 

The study protocol was approved by the Ethics Committee of the Okayama University of Science (No. 27-4, No. 30-2). The study was conducted in accordance with the Declaration of Helsinki. The objective of the study was explained to the participants before starting the study and written informed consent was obtained from each participant. 

### 2.2. Air Sample Collection and Analysis 

Air samples were collected using a mini pump MP-sigma-30NⅡ (flow rate: 0.3 L/min) (Shibata Scientific Technology Ltd., Saitama, Japan) in ORBO-91L absorption tube during both the forest and urban walks (2 h sampling). The ORBO tube samples were extracted into carbon disulfide and analyzed for phytoncides (α-pinene, β-pinene, limonene, *p*-cymene, 3-carene) by gas chromatography–mass spectrometry (GC/MS, JEOL JMS-AMSUN200, Tokyo, Japan). The sample injection volume was 1.0 µL. The analysis conditions were as follows: carrier gas: He, 1.0 mL/min; column: J&W Scientific DB-1 L60 m × 0.25-mm inside diameter, 1.0 mm film); oven temperature program: 40 °C (2 min), and then increasing 7 °C/min to 250 °C (5 min); detector: MSD (a mass selective detector), 220 °C transfer line, SIM (selected ion monitoring) mode. The standard compounds (α-pinene, β-pinene, limonene, *p*-cymene, 3-carene) were purchased from Wako Pure Chemical Industries, Ltd. (Osaka, Japan) and Tokyo Chemical Industry Co., Ltd. (Tokyo, Japan).

### 2.3. Urine Sample Collection and Preparation 

Spot urine (midstream urine) was collected 2–3 days before the walk and the day after the forest or urban walk, and was stored at −80°C until analysis. The samples were centrifuged at 5000 rpm for 5 min at 4 °C to remove cellular fractions and any insoluble materials. Supernatants were used for analysis. All participants were asked not to drink coffee, tea, or alcohol and not to smoke before the urine sampling. Urine sample collections were performed for people who attended both forest and urban walks in 2017–2019, and people who attended the urban walks in 2016.

### 2.4. Determination of Urinary Dityrosine and Hexanoyl-Lysine 

Before analysis, the urine samples were thawed and centrifuged again to remove any insoluble materials. Urinary DT and HEL were quantified by specific competitive enzyme-linked immuno-sorbent assay kits (Dityrosine analysis kit and Hexanoyl-lysine analysis kit, respectively) (Japan Institute for the Control of Aging, Shizuoka, Japan), according to the manufacturer’s protocols. The absorbance was measured at 450 nm with a microplate reader (SH-1200; Corona Electric Co., Ltd., Tokyo, Japan). All measurements were performed in duplicate. 

### 2.5. Determination of Urinary Creatinine 

Creatinine in urine was determined by a commercial alkaline picrate reagent colorimetric assay at 490 nm (R&D Systems, Minneapolis, MN, USA). The values of the urinary DT and HEL were normalized to those of urinary creatinine. Urinary DT and HEL were expressed as micromoles per gram of creatinine and picomoles per milligram of creatinine, respectively.

### 2.6. Data Analysis

All the data are expressed as the mean ± standard error of the mean (SEM). The Kolmogorov– Smirnov test was performed for the normality of urinary DT and HEL data. Because of skewed distribution of urinary DT and HEL levels, they were analyzed by nonparametric statistical method, Mann–Whitney *U* test (pre-walk vs. post-walk), and the level of significance was set at *p* < 0.1 [18]. All statistical analyses were conducted using SPSS Statistic Package version 22 (SPSS Inc., Chicago, IL, USA). 

## 3. Results

### 3.1. Participants’ Characteristics 

Table 1 summarized anthropometric and demographic data. All participants were university students in their twenties (21–23 years old). About 17% (forest walk attendees) or 26% (urban walk attendees) had body mass index (BMI) lower than 18.5 kg/m^2^. Less than 23% were current smokers; less than 30% did habitual exercise/physical activity; about 40% of the participants consumed vegetables regularly and 10% consumed fruits regularly (Table 1). 

### 3.2. Physical Characteristics and Air Phytoncide Levels of the Forest and Urban Environment 

Physical characteristics of the forest and urban fields are shown in Table 2. The minimum and maximum values of air temperature, relative humidity, wind speed, atmospheric pressure, and illuminance of five sites during each walk were given. 

Table 3 shows air phytoncides levels of the forest and urban fields. The total levels of α-pinene, β-pinene, 3-carene, *p*-cymene, and limonene in the forest field were 1.50 times higher compared to those in the urban field, particularly of α-pinene. 

Sampling time for each environment was about 2 h.

### 3.3. Decreased Levels of Urinary DT and HEL after a Forest Walk 

**Figure 2:**
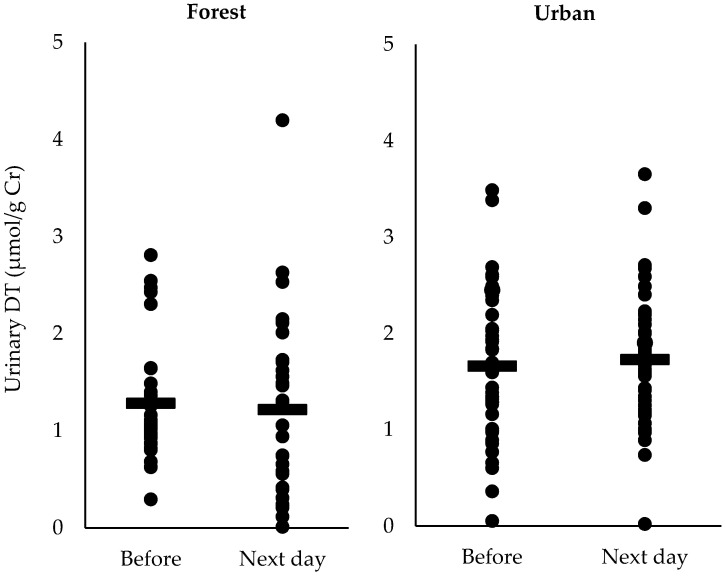
Urinary dityrosine (DT) concentrations pre- and post-forest walk or urban walk. “**―**” denotes the mean value of data.

**Figure 3:**
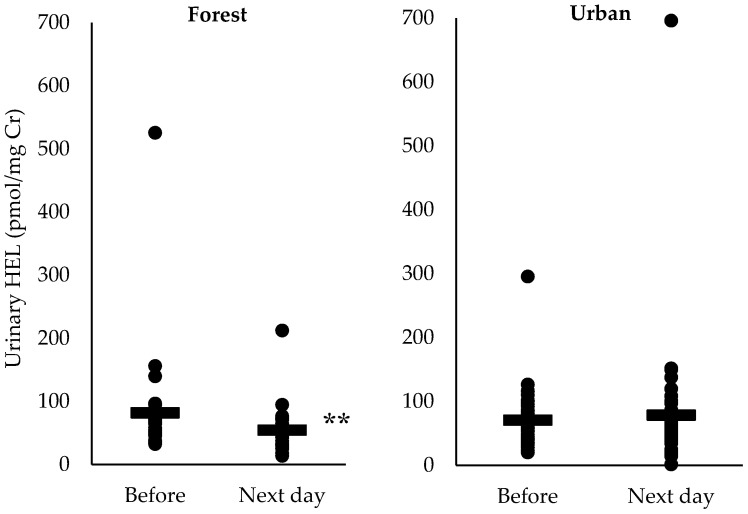
Urinary hexanoyl-lysine (HEL) concentration pre- and post-forest walk or urban walk. “**―**” denotes mean; ** *p* < 0.05: pre-forest walk vs. post-forest walk analyzed by Mann–Whitney *U* test.

Comparison of urinary DT and HEL of pre- and post-walking in each participant who attended the forest and urban walks are shown in Figure 2 and Figure 3, respectively. The concentration of urinary DT in most participants tended to decrease the next day after the forest walk compared with the value pre-walk, although no statistically significant difference was obtained. The concentration of urinary HEL decreased significantly after the forest walk (*p* < 0.05) but not after the urban walk. 

**Figure 4:**
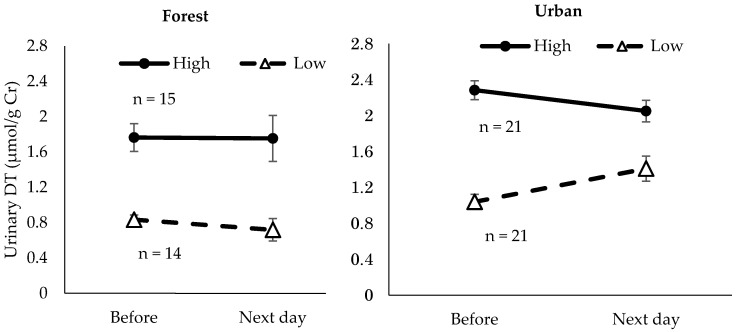
Alteration of urinary DT concentrations in high or low DT group after a forest walk or an urban walk. Data are expressed as mean ± SEM.

**Figure 5:**
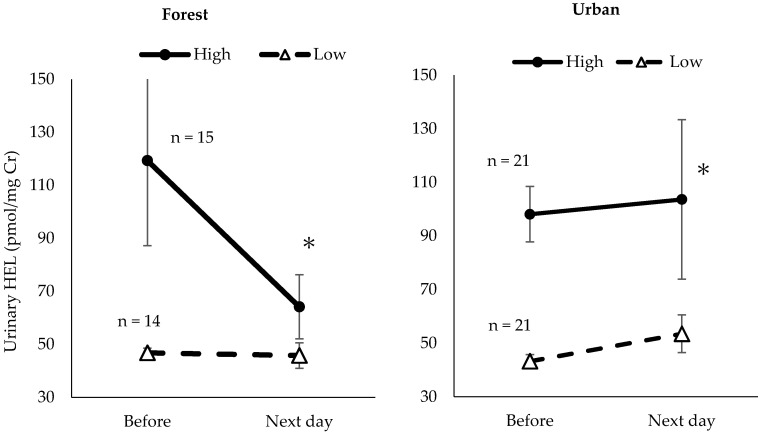
Alteration of urinary HEL concentrations in high or low HEL group after a forest walk or an urban walk. * *p* < 0.1: pre- vs. post-forest walk analyzed by Mann–Whitney *U* test. Data are expressed as mean ± SEM.

We further categorized urinary DT or HEL of pre-walking into 2 groups (high or low)—those whose urinary DT or HEL concentrations of pre-walking were greater than or equal to the median value were categorized as the high group, those whose urine DT or HEL concentrations of pre-walking was lower than the median value as the low group (Figure 4 and Figure 5); we found that the persons who had higher urinary HEL levels of pre-walking showed a significant decrease after the forest walk (*p* < 0.1) and a significant increase after the urban walk (*p* < 0.1). 

## 4. Discussion

Urinary DT and HEL have been proposed to serve as sensitive biomarkers of oxidation of proteins and lipids, respectively [13,14,15,16], and both of them are deleterious products generated by ROS action [11,12]. This pilot report demonstrates, for the first time, a significant decrease in HEL of urine of the participants after a forest walk, but not after an urban walk. The HEL adduct is formed by the reaction of linoleic acid hydroperoxide and lysine residue and is capable of capturing the initial stages of lipid peroxidation in the body [18]. Our results are supported by the findings of Gibo et al. [9], showing a decrease of urinary lipid peroxide level on the next day after a forest walk, although they measured a marker of late-stage lipid peroxidation (MDA-LDL), a useful one for prediction of secondary onset of coronary sclerosis [19]. Some studies showed that the HEL level was significantly higher in the tears of patients with dry eyes [20], and in children with autism spectrum disorders [21]. Kasesawa et al. found a negative correlation between blood HDL cholesterol (‘good cholesterol’) and urinary HEL level, and HEL/adiponectin ratio had a stronger correlation with high sensitivity CRP (an inflammatory indicator) than with adiponectin alone [22], implying HEL may be able to detect oxidative stress in the body, including inflammation. 

In addition, we found the concentration of urinary DT in most participants tended to decrease on the next day after the forest walk compared with the value of pre-walk (Figure 2). Dityrosine release can be considered not only as a marker for protein oxidative damage but also as an endogenous marker for the selective degradation of oxidatively modified proteins [17]. Several reports have demonstrated that DT content was significantly higher in aortic tissue of hyperglycemic animals [23] and that urinary DT excretion was increased in people with diabetes [24] and in children with autism spectrum disorders [25]. 

As shown in Table 3, the relatively high air phytoncides in the forest environment of Shinjo Village compared with that in urban air may partially explain the effect of environmental volatile phytoncides emitted from trees and plants on reducing oxidative stress after entering the body. Some researchers have reported antioxidant and anti-inflammatory effects of phytoncides by attenuation of lipopolysaccharide-induced inflammatory responses through a decrease in oxidative stress production of RAW264.7 macrophage cells and bovine mammary epithelial cells [26,27,28]. We also found some phytoncide compounds (3-carene, limonene, α-pinene, β-pinene) have higher oxygen radical absorbance capacity (data not shown). The research by Sumitomo et al. indicated the blood samples from forest walkers consistently contained α-pinene and the participants’ α-pinene level became 7-fold higher after forest walking [29]. Accordingly, regularly immersing oneself in the forest environment may be expected to prevent oxidative stress-induced disorders by reducing risk of protein and lipid oxidation in biological systems. However, different forest species have different levels of α-pinene, β-pinene, limonene, *p*-cymene, 3-carene etc., so whether the positive effects are because of being in the forest (regardless of which trees are present) or if they are based on the levels of phytoncides requires more evidence, which is expected to prove such a relation in future study. 

Although this is the first report of decreased urinary DT and HEL levels after a forest walk, the current study does have some limitations: (1) the study sample was small and the participants were senior university students which limits generalization of the findings; in addition, the difference in number of participants in forest walks and urban walks may introduce some variability in urban walks comparatively to forest walks; (2) the level of statistical significance in pre-post comparisons of urinary DT and HEL was set at *p* < 0.1, which is higher than the conventional cutoff of 0.05, because of the small sample size and high variability in urinary levels of DT and HEL, and this might mean only small-study effects can be detected at this level. 

## 5. Conclusions

This work shows evidence of decreased urinary DT and HEL excretion after a forest walk and of relatively high air phytoncides in the forest environment as well, suggesting the possibility that regular immersion in a forest environment, even for a short time, might contribute toward weakening of the oxidative modifications of proteins or lipids in the body. Assessing urinary DT and HEL at pre- post-forest walk in a large number of individuals is a major goal of future research. Moreover, future studies focusing on the evaluation of other biomarkers related to protein and lipid oxidation would support the present results and be helpful in understanding the mechanisms of the health benefits of forest environments. 

## Figures and Tables

**Table 1:** Anthropometric and Demographic Data.

Variable	Forest (Shinjo Village)			Urban		
	Total (n = 29)	Male (n = 22)	Female (n = 7)	Total (n = 42)	Male (n = 31)	Female (n = 11)
	n (%)	n (%)	n (%)	n (%)	n (%)	n (%)
BMI (kg/m^2^)						
<18.5	5 (17.2)	4 (18.2)	1 (14.3)	6 (26.2)	4 (12.9)	2 (18.2)
≥18.5	24 (82.8)	18 (81.8)	6 (85.7)	36 (73.8)	27 (87.1)	9 (81.8)
Smoker						
No	25 (86.2)	18 (81.8)	7 (100.0)	34 (81.0)	24 (77.4)	10 (90.9)
Present	4 (13.8)	4 (18.2)	−	7 (16.6)	7 (22.6)	−
Past	−	−	−	1 (2.4)	−	1 (9.1)
Alcohol Drink						
No	13 (44.8)	10 (45.5)	3 (42.9)	22 (52.4)	17 (54.8)	5 (45.5)
<3 times/week	16 (55.2)	12 (54.5)	4 (57.1)	18 (42.9)	12 (38.7)	6 (54.5)
≥4 times/week	−	−	−	2 (4.7)	2 (6.5)	−
Habitual Exercise/Physical Activity						
No	24 (82.8)	17 (77.3)	7 (100.0)	34 (81.0)	23 (74.2)	11 (100.0)
Yes	5 (17.2)	5 (22.7)	−	8 (19.0)	8 (25.8)	−
Vegetable Intake						
Almost none	3 (10.3)	2 (9.1)	1 (14.3)	2 (4.8)	2 (6.5)	−
Sometimes	11 (37.9)	9 (40.9)	2 (28.6)	22 (52.4)	17 (54.8)	5 (45.5)
A little per day	13 (44.8)	10 (45.5)	3 (42.8)	17 (40.4)	11 (35.5)	6 (54.5)
A lot per day	2 (6.9)	1 (4.5)	1 (14.3)	1 (2.4)	1 (3.2)	−
Fruit Intake						
Almost none	9 (31.0)	8 (36.4)	1 (14.3)	12 (28.6)	10 (32.3)	2 (18.2)
Sometimes	15 (51.7)	10 (45.4)	5 (71.4)	24 (57.1)	16 (51.6)	8 (72.7)
A little per day	3 (10.3)	2 (9.1)	1 (14.3)	5 (11.9)	4 (12.9)	1 (9.1)
A lot per day	2 (6.9)	2 (9.1)	−	1 (2.4)	1 (3.2)	−

**Table 2:** Physical characteristics of the forest and urban environment.

Variable	Forest Environment	Urban Environment
Air temperature (°C)	14.8–23.8	22.2–36.3
Relative humidity (%)	56.1–94.3	34.2–77.7
Atmospheric pressure (hPa)	919–930	889–1018
Illumination (lx)	54–3384	421–52,800
Wind speed (m/s)	0.08–1.37	0.02–2.67
Elevation (m)	700 ± 50	2–7

**Table 3:** Air phytoncides in the forest and urban environments.

Variable	Forest Environment	Urban Environment
α-Pinene (μg/m^3^)	15.15	5.74
β-Pinene (μg/m^3^)	1.69	1.29
3-Carene (μg/m^3^)	7.03	5.06
*p*-Cymene (μg/m^3^)	12.44	8.94
Limonene (μg/m^3^)	32.57	24.84
Total	68.87	45.87
